# Cellular and Viral Factors Regulating Merkel Cell Polyomavirus Replication

**DOI:** 10.1371/journal.pone.0022468

**Published:** 2011-07-22

**Authors:** Huichen Feng, Hyun Jin Kwun, Xi Liu, Ole Gjoerup, Donna B. Stolz, Yuan Chang, Patrick S. Moore

**Affiliations:** 1 Cancer Virology Program, University of Pittsburgh Cancer Institute, University of Pittsburgh, Pittsburgh, Pennsylvania, United States of America; 2 Department of Cell Biology and Physiology, Center for Biologic Imaging, University of Pittsburgh, Pittsburgh, Pennsylvania, United States of America; University of Kansas Medical Center, United States of America

## Abstract

Merkel cell polyomavirus (MCV), a previously unrecognized component of the human viral skin flora, was discovered as a mutated and clonally-integrated virus inserted into Merkel cell carcinoma (MCC) genomes. We reconstructed a replicating MCV clone (MCV-HF), and then mutated viral sites required for replication or interaction with cellular proteins to examine replication efficiency and viral gene expression. Three days after MCV-HF transfection into 293 cells, although replication is not robust, encapsidated viral DNA and protein can be readily isolated by density gradient centrifugation and typical ∼40 nm diameter polyomavirus virions are identified by electron microscopy. The virus has an orderly gene expression cascade during replication in which large T (LT) and 57kT proteins are first expressed by day 2, followed by expression of small T (sT) and VP1 proteins. VP1 and sT proteins are not detected, and spliced 57kT is markedly diminished, in the replication-defective virus suggesting that early gene splicing and late gene transcription may be dependent on viral DNA replication. MCV replication and encapsidation is increased by overexpression of MCV sT, consistent with sT being a limiting factor during virus replication. Mutation of the MCV LT vacuolar sorting protein hVam6p (Vps39) binding site also enhances MCV replication while exogenous hVam6p overexpression reduces MCV virion production by >90%. Although MCV-HF generates encapsidated wild-type MCV virions, we did not find conditions for persistent transmission to recipient cell lines suggesting that MCV has a highly restricted tropism. These studies identify and highlight the role of polyomavirus DNA replication in viral gene expression and show that viral sT and cellular hVam6p are important factors regulating MCV replication. MCV-HF is a molecular clone that can be readily manipulated to investigate factors affecting MCV replication.

## Introduction

Merkel cell polyomavirus (MCV) was identified by digital transcriptome subtraction from Merkel cell carcinoma (MCC), a rare but aggressive human skin cancer [1], [2]. MCV is a double-stranded DNA virus belonging to the *Polyomaviridae* family, members of which share conserved early, late, and regulatory regions. The polyomavirus early viral tumor (T) antigens play key roles in viral genome replication as well as tumorigenesis. Large T (LT) antigen-encoded helicase activity, for example, unwinds the viral replication origin [3], [4] and enhances the polyomavirus late promoter leading to an early-to-late switch in gene expression. For murine polyomavirus, this switch has been shown to depend on LT-initiated viral DNA replication [5]. The late region encodes viral capsid proteins (VP1 and VP2) that self-assemble into virus-like particles (VLP) when expressed in cells [6], [7], [8], [9], [10]. MCV VLP have been used to infect cells and can be used in neutralization experiments [6] but replication of full MCV genome has not been described. The concerted regulation and interaction of both early and late polyomavirus proteins are necessary to produce viral particles.

Loss of viral replication capacity, or permissivity, is a common feature of virus-initiated tumors [11], [12]. Approximately 80% of MCC are infected with MCV in which the viral genome is clonally-integrated into the host tumor cell genome, preventing viral replication [1], [13], [14]. MCV obtained from tumors also possess LT gene mutations that are a central feature of MCV-driven human tumor formation [13]. LT normally binds a specific site in the viral replication origin and initiates DNA replication through its C-terminal helicase domain. Tumor-specific mutations prevent LT-initiated DNA replication at the integrated genome thus preventing independent and unlicensed DNA replication from the viral genome that could lead to catastrophic replication fork collisions and DNA breakage when multiple virus-initiated replication forks proceed onto the cellular DNA template [13].

The minimal MCV replication origin has been mapped to a 71 bp fragment in a non-coding region that LT protein binds in order to initiate viral DNA replication. Among MCV proteins, LT protein alone is sufficient for this process but MCV small T (sT) protein acts as an accessory factor that greatly increases the efficiency of MCV origin firing [3]. We have recently described MCV sT as a transforming oncoprotein in MCC that inactivates the Akt-mTOR pathway protein 4E-BP1 and activates cap-dependent protein translation in MCV-positive tumors [15]. In one MCV tumor strain (MCV350), a point mutation in its replication origin prevents proper assembly of the LT helicase complex, also rendering the tumor-derived virus nonpermissive [3]. Additional virus mutations in capsid genes, including in the MCV350 strain, have been described that are predicted to prevent virion self-assembly and replication [16]. The sT and the N-terminal portions of LT, however, are unaffected by tumor-specific mutations, suggesting that they may play a key role in MCC tumorigenesis. The importance of viral early gene contributions to this cancer are shown by knockdown of the common T antigen exon 1 sequence, which leads to cell cycle arrest and cell death of MCV-positive MCC cells [17].

More than 50% of the healthy adult population is serologically positive for MCV antibodies [6], [7], [18], [19] and most adult MCV infections are asymptomatic [20]. In contrast to MCC tumors, only very low level MCV genomic DNA is present in healthy tissues, including skin, peripheral blood mononuclear cells, gastrointestinal tract [1], [21], [22], human respiratory tract secretions [23], and other tissues [21]. Using rolling circle amplification, Schowalter et al. have recently isolated several encapsidated strains of wild-type MCV from healthy skin [22]. Nonetheless, the limiting amounts of wild-type MCV flora present in healthy tissues have been a significant barrier to isolation of replication-competent MCV.

To search for novel cellular factors binding to MCV early proteins, we performed tandem-affinity pulldown assays with a unique region of the MCV LT [24]. An MCV LT domain that is conserved in both tumor-derived and wild-type MCV strains interacts with the cytoplasmic vacuolar sorting protein, hVam6p (also known as Vps39), a component of the HOPS (homotypic fusion and protein sorting) complex involved in late endosomal and lysosomal fusion [25]. Coexpression of MCV LT with hVam6p causes relocalization of hVam6p from the cytoplasm, where it is normally found, to the nucleus and to perinuclear bodies. This interaction can be abrogated by a single alanine substitution at tryptophan 209 (LT.W209A) in the hVam6p binding domain of LT. No differences in cell viability or cell cycling have been detected for the wild-type LT and LT.W209A expression and so the role for this LT interaction remains unknown. MCV LT antagonizes the ability of hVam6p to induce lysosomal clustering, raising the possibility that hVam6p might modulate MCV replication or egress [24].

Development of an MCV molecular clone allows engineering the viral genome, such as introducing a mutation at the hVam6p interaction site in LT, to examine effects on virus replication and assembly. To generate an MCV clone, we aligned MCV genomes from MCC tumors as well as non-tumor tissues and designed a consensus MCV molecular genome (MCV-HF). Subsequent to construction of this consensus clone, the same viral sequence was identified in several naturally occurring MCV strain obtained from healthy human skin samples [22], supporting the notion that MCV-HF is a permissive viral clone.

We show here that the MCV-HF clone is replication competent and sequentially expresses early and late viral proteins to generate packaged virions after transfection of the molecular clone DNA into 293 cells. MCV-HF has modest but reproducible replication capacity in a variety of tissue culture cell lines that is enhanced by MCV sT coexpression. In contrast, MCV engineered with the MCV350 origin point mutation is replication-deficient, only transiently expresses LT protein, has diminished or absent expression of other early spliced transcript protein forms and does not express late MCV protein. Overexpression of hVam6p is potent in inhibiting MCV replication whereas engineering MCV-HF with the LT.W209A mutation amplifies virus replication suggesting a key role for this vacuolar sorting protein in MCV virion production.

## Results

### Design and construction of a consensus MCV genome

**Figure 1:**
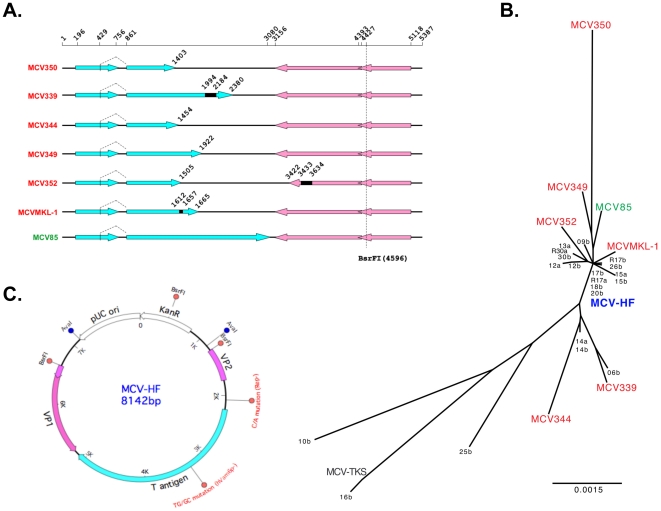
MCV genome. (**A**) Full-length of MCV genomes identified from 5 MCC tumors (MCV350, MCV339, MCV344, MCV349, MCV352), 1 MCC cell line (MCVMKL-1) and 1 PBMC sample (MCV85). T antigen ORFs are shown in blue arrows, VP ORFs in pink arrows. Numbers stand for positions in MCV genome. Black solid boxes indicate genomic deletions in MCV genome. (**B**) Phylogenetic tree of MCV genomes. The consensus genome (MCV-HF, JF813003) is located in the center of a tree including MCV350 (EU375803), MCV339 (EU375804), MCV344 (JF812999), MCV349 (JF813000), MCV352 (JF813001), MCVMKL-1 (FJ173815), MCV85 (JF813002) and other MCV sequences obtained from human skin [22] and Kaposi's sarcoma [40]. (**C**) The consensus MCV-HF genome can be linearized at *BsrF*I site (4,596 nt) and cloned for propagation in *E. coli*. Sites for mutations engineered into two MCV-HF genomes (MCV-Rep^−^ (C/A) and MCV-hVam6p^−^ (TG/GC)) are shown.

At the initiation of this study, no full-length viral genomes from nontumor sources had been isolated. We originally found 8 of 10 (80%) MCC tumors to be positive for MCV DNA [1]. We sequenced full-length MCV genomes by PCR-direct sequencing from 5 of the 8 virus-positive tumors (MCV350, MCV339, MCV344, MCV349, MCV352), as well as from 1 MCV positive cell line (MKL-1), which were compared to a single whole virus sequence (MCV85) obtained from a peripheral blood sample (Fig. 1A). All tumor-derived sequences have T antigen truncations, including mutations or short genomic deletions. For some strains, tumor-derived mutations are also observed in late gene regions, as reported by A. Zur Hausen's group [16]. For example, a 200-bp deletion is present in the VP1 locus of MCC352, generates a truncated VP1 protein that is likely to lead to incomplete viral assembly for this strain (Fig. 1A).

To address the issue of polymorphisms between and within individual cases, we designed a consensus genome (MCV-HF, *GenBank ID: JF813003*). This cloned genomic DNA (available through our website, www.tumorvirology.pitt.edu/mcvtools.html) is based on the 7 full-length MCV genomes. Compared to these genomes, the MCV-HF genome is located centrally in the phylogenetic tree (Fig. 1B), closest to wild-type R17a strain and has the same nucleotide sequence as the 17b, 18b and 20b strains identified by Schowalter et al. from human normal skin [22]. The consensus MCV-HF was synthesized and cloned into a kanamycin selectable vector (Fig. 1C). An MCV genome variant possessing the MCV350 mutation in the replication origin (MCV-Rep^−^) was generated by site-directed mutagenesis to serve as a negative control for viral replication [3].

### MCV-HF and MCV-Rep^−^ viral protein expression in 293 cells

**Figure 2:**
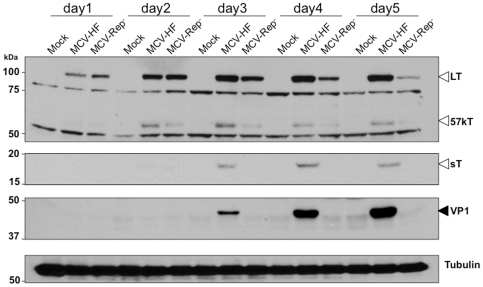
Coordinated viral gene expression during MCV replication in 293 cells. One microgram of recircularized MCV genomes (wild-type MCV-HF or replication-defective MCV-Rep^−^) was transfected into 293 cells. Immunoblotting was performed to examine T antigen expression over 5 days (indicated by hollow arrows) and VP1 protein (indicated by solid arrow) using CM2B4 (LT, 57kT), CM8E6 (sT) and CM9B2 (VP1) antibodies, respectively. Alpha-tubulin detection was used as a protein loading control. LT protein is expressed equally at day 2 for both viruses but decreases for MCV-Rep^−^ on days 3–5. VP1 increases on days 3–5 only for MCV-HF, corresponding to viral DNA replication (Fig. 3D). Other early proteins are also diminished (57kT) or absent (sT) in the replication deficient MCV-Rep^−^.

MCV-HF or MCV-Rep^−^ circular genome DNAs were transfected into 293 cells and viral protein expression was determined by immunoblotting for LT, 57kT, sT antigen and VP1 proteins (Fig. 2). The defective replication origin in MCV-Rep^−^ genome does not affect initial LT antigen expression, indicating that this mutation does not directly alter early gene transcription. LT protein is readily detected 24 hrs after transfection for both MCV-HF and MCV-Rep^−^ and equal amounts of LT expressed by day 2 for both clones. This reveals that the early promoter regulating LT is intact in both viruses and similarly active at time points prior to active viral replication and amplification. Surprisingly, expression of the alternatively spliced 57kT form is reduced with MCV-Rep^−^ at day 2. This is not due to differences in detection since both LT and 57kT are determined on the same blots with the same CM2B4 antibody. LT protein expression continues over 5 days for the MCV-HF virus. LT protein levels peak at 48 hrs for MCV-Rep^−^ and then subsequently decline. Notably, the spliced 57kT antigen protein is diminished at all time points for the MCV-Rep^−^ clone. In contrast to LT and 57kT, no sT (an alternate spliced form from the early Tag locus) expression is detected after transfection of MCV-Rep^−^, but sT is readily detected by day 3 after transfection with the replication-competent genome. These findings suggest that splicing efficiency among early MCV genes may be dependent on viral genome replication.

VP1 structural protein also increases in abundance from day 3 through 5 post MCV-HF transfection (Fig. 2), consistent with a switch from early to late gene expression to generate spontaneously assembling virus particles. VP1 expression, however, is not detected after MCV-Rep^−^ transfection. Thus, in 293 cells, transient transfection of only the wild-type MCV-HF genome produces both early and late proteins required for virus replication and assembly. Alternatively-spliced early proteins (sT and 57kT) are diminished or absent, as is the late VP1 protein, for the MCV-Rep^−^ virus despite similar initial levels of LT expression with both permissive and nonpermissive viruses.

### Detection of MCV-HF virus in 293 cells

**Figure 3:**
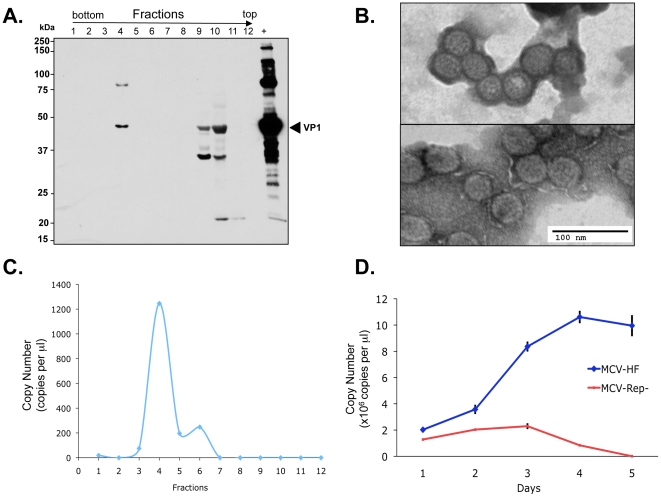
Fractionation of viral capsid protein VP1 by Optiprep™ density gradient ultracentrifugation. (**A**) Twelve fractions were collected from highest to lowest density, and analyzed by immunoblotting with CM9B2 antibody to detect VP1 capsid protein. Assembled 45 kDa VP1 protein is isolated in fraction 4. Unassembled, free VP1 protein is present in Fractions 9 and 10. The positive control (+) is virus-like particle (VLP) prepared from 293TT cells by MCV VP1 and VP2 transfection. (**B**) Typical 40 nm diameter icosahedral Merkel cell polyomavirus particles present in fraction 4 (upper panel) and VP1/VP2-containing MCV virus-like particles for comparison (bottom panel). (**C**) Nuclease-resistant MCV DNA in various gradient fractions quantitated by real time PCR. Highest levels of encapsidated DNA are present in fraction 4, corresponding to the fraction having MCV virions. (**D**) Time-course for MCV virion production after transfection of 1 µg replication competent (MCV-HF) or incompetent (MCV-Rep^−^) genomes into 293 cells was determined on lysed cells by quantitative PCR after nuclease treatment. Genome replication and packaging of MCV-HF is evident by day 3.

We next examined virion production in 293 cells transfected with MCV-HF. At day 4 post MCV-HF transfection, cells were harvested, lysed, matured overnight and treated with benzonase, RNase A and Plasmid-safe™ nuclease. Fractions were collected from an ultracentrifuged Optiprep™ (iodixanol) gradient and immunoblotted for VP1 protein (Fig. 3A). High molecular weight aggregates of the ∼45 kDa VP1 protein are present in fraction 4 having a 1.24 g/ml buoyant density. A high molecular weight VP1 form (∼90 kDa) is also present polyacrylamide gels that may represent covalently-crosslinked dimeric VP1 protein. VP1 is also present in the lowest density fractions 9 and 10, that represent unassembled, free VP1 protein. Fraction 4 contains typical 38–43 nm diameter polyomavirus particles detected by transmission electron microscopy with uranyl acetate negative staining (Fig. 3B). Quantitative real-time PCR for DNA isolated from Optiprep gradients reveals highest copy numbers of nuclease-resistant DNA in fraction 4 (Fig. 3C). In contrast, nuclease-resistant MCV genome is not detected in fractions 9 and 10, consistent with digestion of unencapsidated DNA. Encapsidated viral DNA increases up to day 4 after MCV-HF transfection and then plateaus (representative results are shown in Fig. 3D), correlating with VP1 protein expression levels. As expected for the nonreplicating virus, viral DNA is present immediately after MCV-Rep^−^ transfection but diminishes below the level of detectability by day 5.

**Figure 4:**
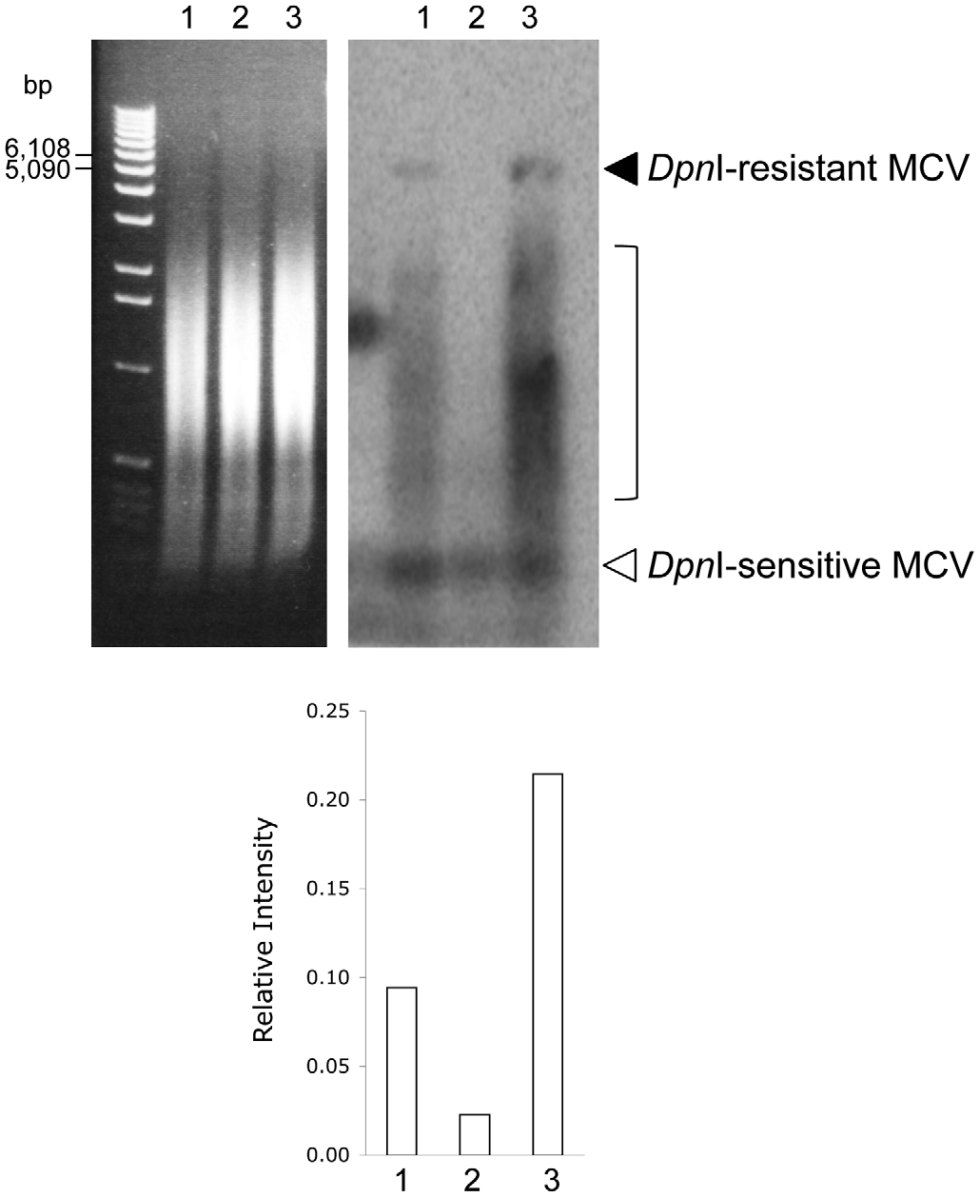
MCV genome replication. Southern blot (right panel) for MCV for MCV-HF (lane 1), MCV-Rep^−^ (lane 2) and MCV-hVam6p^−^ (lane 3) viruses four days after transfection of 1 µg circular genomic DNA into 293 cells. Panel on left shows the ethidium bromide-stained gel prior to transfer indicating equal DNA loading. Bands for the full-length 5.4 kb MCV genome are present as *Dpn*I-resistant bands in MCV-HF and MCV-hVam6p^−^ viruses (lanes 1 and 3) but not in the replication deficient MCV-Rep^−^ virus (lane 2). The replication efficiency was measured by the ratio between the *DpnI*-resistant 5.4 kb band and the *DpnI*-sensitive band. The MCV-hVam6p^−^ virus generates ∼2-fold more full length genome compared to wild-type MCV-HF virus. Replicated viral DNAs also show the presence of extensive subgenomic fragments.

These findings are confirmed by Southern blotting for viral DNA (Fig. 4). In this experiment, DNA from 293 cells 4 days post-transfection, with either MCV-HF (lane 1) or MCV-Rep^−^ (lane 2), were treated with *Dpn*I to digest unreplicated DNA and with *BamH*I to linearize MCV genome. No bands are present for MCV-Rep^−^, while a weak 5.4 kb *Dpn*I-resistant band representing full-length genome is present in extracts from MCV-HF transfected 293 cells. Significantly, a subgenomic 0.3–2 kb smear of *Dpn*I-resistant MCV DNA is also present, consistent with a large fraction replicated MCV DNA being composed of either abortively-replicated or partially-digested genome fragments.

### Optimization of MCV clone replication

**Figure 5:**
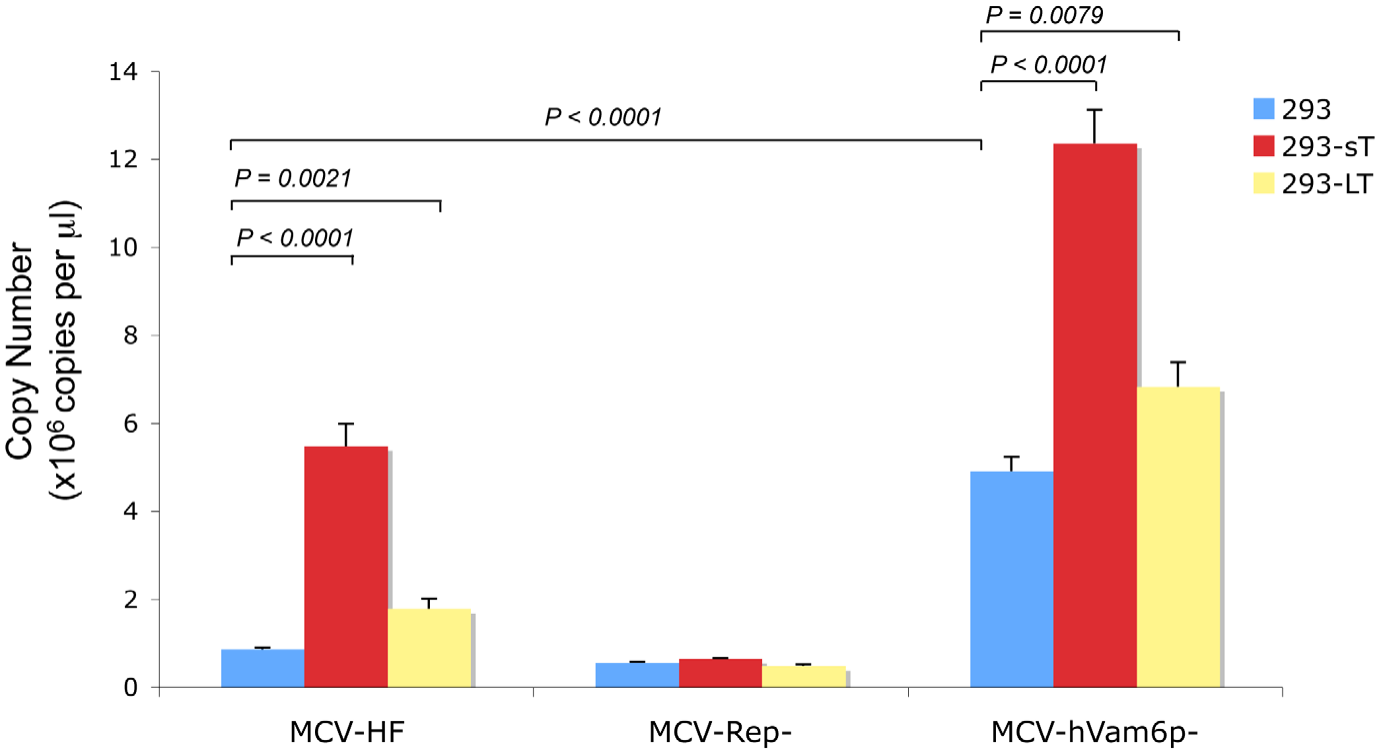
Quantitative PCR for MCV virion production for MCV-HF, MCV-Rep^−^ and MCV-hVam6p^−^ viruses. One microgram MCV clone DNAs were transfected into 293 cells stably transduced to express MCV sT or LT proteins (not shown). DNA was extracted and treated with benzonase and RNase to discriminate packaged viral DNA. The nuclease-resistant MCV genome was precipitated and measured by quantitative PCR after proteinase K treatment. Cellular sT expression increases virion production for both MCV-HF and MCV-hVam6p^−^ viruses. Comparison of MCV-HF and MCV-hVam6p^−^ shows that loss of the hVam6p binding site also increases virus production. Coexpression of sT and mutation of the hVam6p binding site in the MCV genome are additive in virion production compared to MCV-HF without sT coexpression.

To determine whether coexpression of MCV early genes might enhance MCV replication, 293 cells were stably transduced with MCV sT, LT or empty vector expression constructs. MCV-HF and MCV-Rep^−^ genomes were then transfected into these cells and MCV virion production was quantitated by real-time PCR. Cells coexpressing MCV sT generate approximately 5-fold increased nuclease-protected MCV DNA compared to cells without sT coexpression (Fig. 5). In contrast, only a small increase in MCV DNA is present in cells stably expressing LT protein, suggesting that sT but not LT protein levels are limiting for genome replication after transfection. Several other cell lines (UISO, 293TT, NIH3T3 and COS7) were also examined, with and without various MCV gene coexpressions, in an attempt to optimize MCV virion production (Table 1). None of these conditions led to appreciably greater MCV virion production compared to 293 cells alone.

**Table 1:** Optimization of MCV production in various cell lines and effect of co-expression of viral proteins.

Cell lines	Co-transfected MCV plasmid(s)	Production level*
293	None^§ ¶^	++
	Large T antigen^§ ¶^	++
	Small T antigen^§ ¶^	+++
UISO	None^§^	+
	Genomic T antigen^§^	+
	VP1 and VP2^§^	+
293TT	None^§^	+/−
	Genomic T antigen^§^	+
	VP1 and VP2^§^	+/−
3T3	None^§^	+/−
	Genomic T antigen^§^	+
COS7	None^§^	+/−
	Genomic T antigen^§^	+

*Note: Relative expression was determined in individual experiments by PCR(§) , MCV protein expression (¶), or both.

### hVam6p interaction with MCV LT diminishes MCV replication

**Figure 6:**
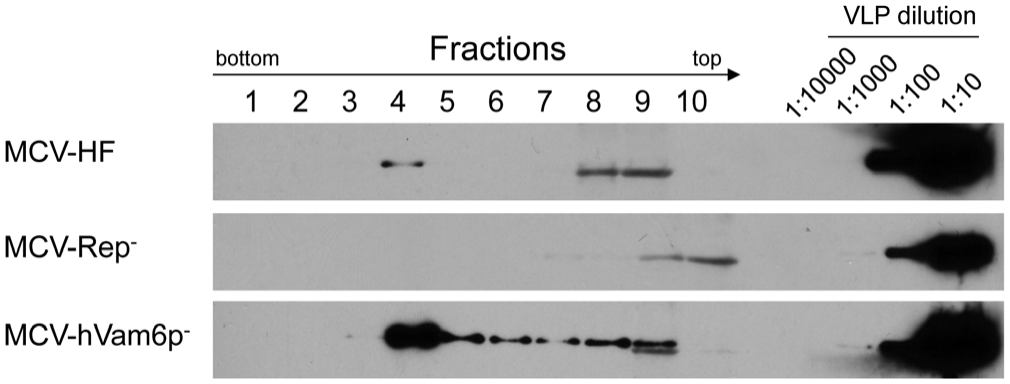
Comparison of viral packaging for MCV-HF, MCV-Rep^−^ and MCV-hVam6p^−^ viruses. Optiprep™ density gradient fractions from wild type (MCV-HF) and mutant viruses (MCV-Rep^−^, MCV-hVam6p^−^) generated from transfected 293 cells were used for Western blotting. Dilutions of MCV virus-like particles (VLP) provide a marker for the relative abundance of VP1 protein in each fraction. Assembled MCV-hVam6p^−^ virus VP1 expression is ∼10 fold increased in fraction 4 compared to MCV-HF.

**Figure 7:**
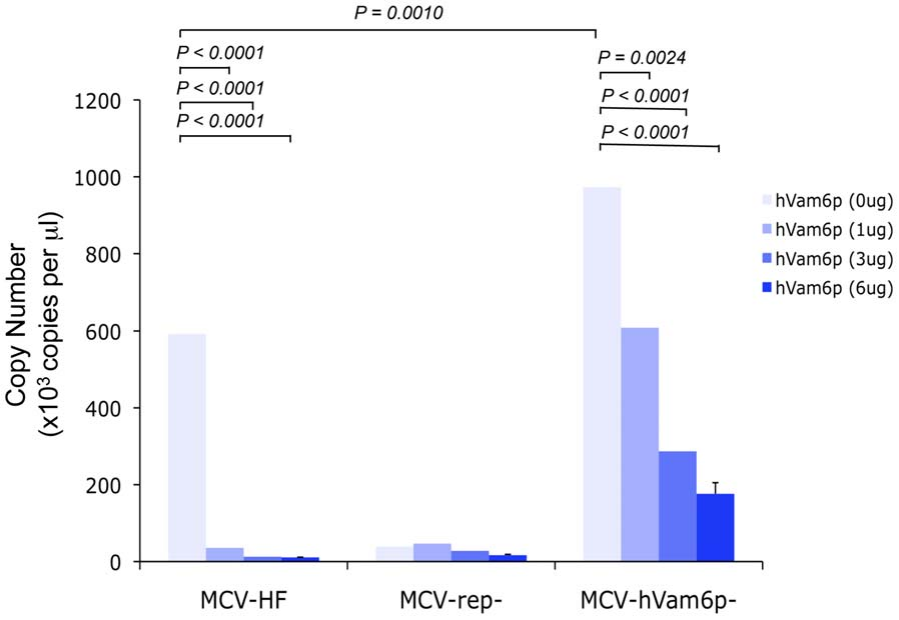
Effect of hVam6p coexpression on MCV virion production. 293 cells cotransfected with hVam6p and MCV genomes at day 4 as measured by nuclease-resistant DNA by quantitative PCR. Circularized viral plasmids (1 µg) together with varying amounts of hVam6p expression plasmid were simultaneously transfected during this experiment.

Tandem-affinity pulldown studies have found that MCV LT binds to hVam6p and relocalizes it from the cytoplasm to the nucleus, but the biological importance of this interaction is unknown [24]. A mutant MCV-HF, designated as MCV-hVam6p^−^, was engineered to encode a W209A substitution in the LT gene to prevent hVam6p interaction and to test its role in MCV replication. As seen in Fig. 5, mutation of the hVam6p-binding site leads to a 4–6 fold increase in nuclease-resistant virion production compared to the wild-type MCV-HF virus. Enhanced virion replication is additive with sT coexpression, suggesting that LT.W209A and sT coexpression are independent of each other and act at different stages of replication. Increased virion and subgenomic DNA production for MCV-hVam6p^−^ was confirmed by Southern blotting (Fig. 4, lane 3). The comparable levels of *Dpn*I-sensitive bands in all lanes on the Southern blot demonstrate that this effect is unlikely to be due to differences in transfection efficiency. Immunoblotting for encapsidated VP1 after gradient purification also reveals markedly increased viral particle production with MCV-hVam6p^−^ genome (Fig. 6). In contrast, hVam6p coexpression inhibits nuclease-resistant, encapsidated DNA production from MCV-HF to levels comparable to the nonpermissive MCV-Rep^−^ virus (Fig. 7). The MCV-hVam6p^−^ clone replication is relatively resistant to the effects of hVam6p coexpression compared to MCV-HF but also declines in a dose-dependent fashion. hVam6p overexpression does not decrease 293 cell viability or alter cell cycling, nor does it alter transfection efficiency (data not shown).

**Figure 8:**
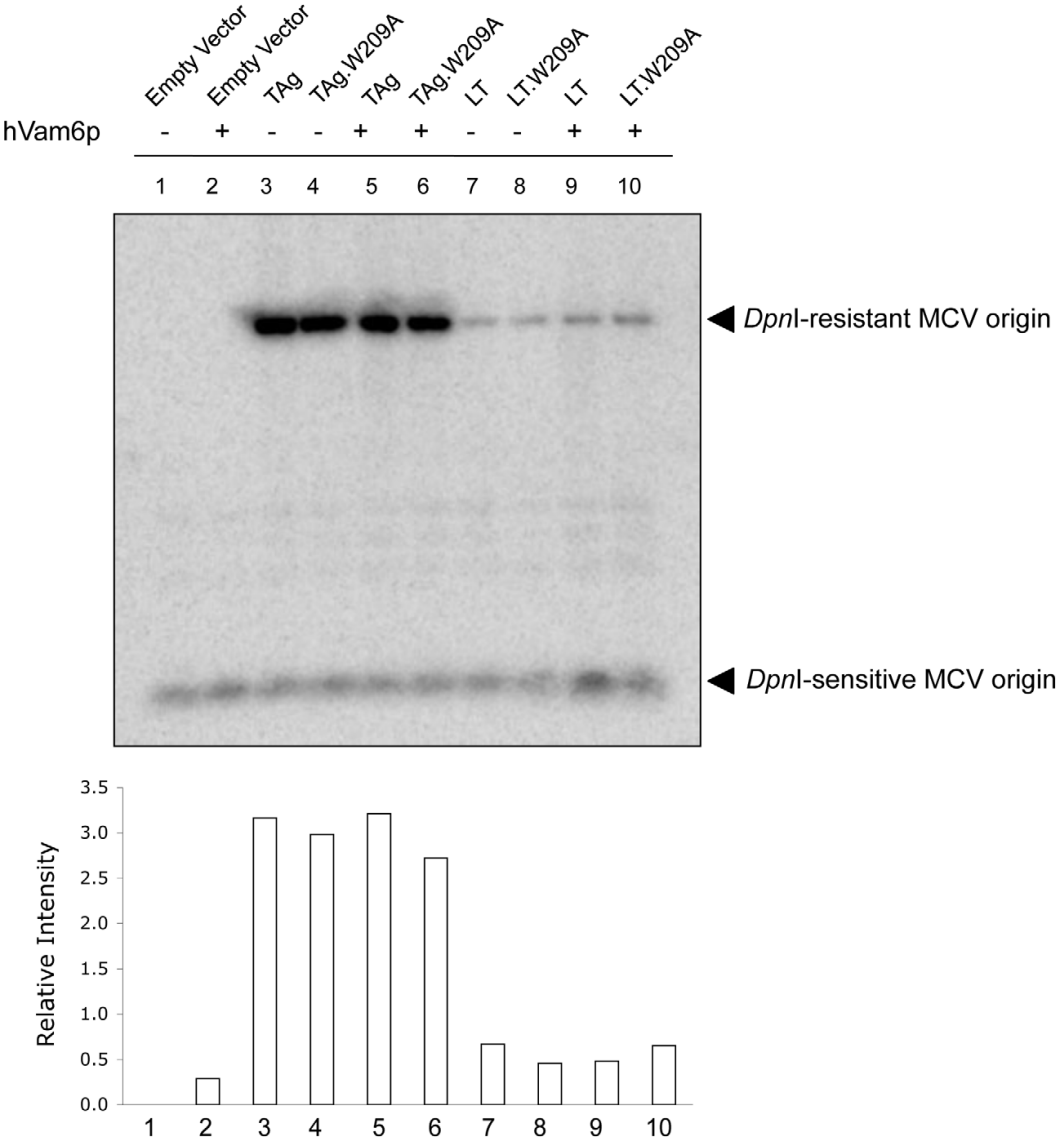
Effects of hVam6p on *in vitro* MCV origin replication. 293 cells were transfected with plasmids containing MCV origin and equal amounts of either wild type of genomic T antigen (TAg), LT cDNA, or the corresponding constructs containing the hVam6p-binding site mutations (TAg.W209A and LT.W209A). Origin replication was assessed through Southern blotting by comparing the ratio of *Dpn*I-resistant (replicated) to *Dpn*I-sensitive (unreplicated) DNA. For each condition, replication in the absence or presence of simultaneously cotransfected hVam6p expression plasmid was determined. Expression of the genomic TAg containing both sT and LT showed increased replication of the MCV origin compared to the LT cDNA regardless of hVam6p coexpression. Neither mutation of the hVam6p binding site on LT nor coexpression of exogenous hVam6p significantly altered MCV origin replication.

Since hVam6p is normally a cytoplasmic protein that relocalizes to nuclear and perinulclear sites when MCV LT is co-expressed, we sought to determine whether hVam6p might directly inhibit viral DNA synthesis in the presence of LT (Fig. 8). An *in vitro* origin replication assay was performed with either the entire T antigen gene locus, TAg (expressing LT, 57kT and sT), or the LT cDNA alone. These plasmids were transfected together with the MCV origin cloned into the pCR2.1 vector, with or without hVam6p expression. Southern blotting for the origin plasmid measures T antigen-initiated DNA replication (*Dpn*I-resistant band) relative to unreplicated, transfected origin DNA (*Dpn*I-sensitive band) [3].

As previously reported [3], the genomic TAg locus (Fig. 8 , lane 3) expressing all early proteins is markedly more efficient at initiating origin replication than the LT cDNA alone (Fig. 8, lane 7), consistent with an accessory role for sT in LT-mediated DNA replication. Unlike virion production, however, coexpression of hVam6p does not significantly change *in vitro* MCV origin replication by TAg (Fig. 8, lane 5) or LT cDNA (Fig. 8, lane 9). Further, the LT.W209A substitution in either TAg or LT cDNA does not appreciably affect origin replication efficiency (Fig. 8, lanes 4, 6, 8 and 10).

### Failure to achieve secondary MCV-HF transmission

We used fraction 4 (Fig. 3A), which contains encapsidated MCV virions to infect a variety of cell types including 293, 293TT, UISO, A549, BJAB, Raji, BSC40 and PBMC purified from whole blood with or without polybrene treatment. No cytopathic effect (CPE) was observed in long-term culture (4 weeks). Immunoblotting and immunofluorescence staining for T antigen and VP1 proteins were not positive for cells exposed to virus (data not shown). We also did not detect viral transcripts by RT-PCR (not shown), suggesting no secondary detectable infection occurred. Co-culture of MCV-HF transfected 293 cells with 293, A549 and UISO cells separated by 0.2 or 0.45-µm membranes on a transwell plate also failed to demonstrate secondary virus infection. These results may be due in part to the limited amount of infectious virus generated after transfection.

## Discussion

We generated a replicating MCV molecular clone that can be manipulated to assess effects on virus DNA replication and encapsidation. As previously seen in origin-replication studies [3], introduction of the MCV350 strain point mutation into the MCV-HF replication origin abolishes the clone's ability to replicate. We also confirm that MCV sT protein expression together with LT expression is required for optimal MCV replication, a feature of viral genome replication that MCV shares with the human JC polyomavirus [26].

Fully-encapsidated MCV virions from MCV-HF were isolated in fraction 4 at 1.24 g/ml on iodixanol (Optiprep™) gradients. Evidence that these are fully-encapsidated viruses include isolation of nuclease-protected DNA and ultra-high molecular weight aggregates of VP1 protein specific to this fraction. In the 1970s, hyperosmolar CsCl isopycnic gradients were used to isolate polyomaviruses having higher apparent buoyant densities (e.g., 1.34 g/ml [27]). It is now well-established that hyperosmolar CsCl gradients overestimate the densities of large macromolecular structures, such as viruses, since CsCl gradients dehydrate virions, replacing water with heavy salts, which reduces viability and artifactually increases the buoyant density. JC virus has a buoyant density of 1.20 g/ml on linear sucrose gradients and 1.35 g/ml in CsCl gradients [28]. Similarly, goose hemorrhagic polyomavirus virion has a 1.20 g/ml density in sucrose and an apparent 1.34–1.35 g/ml density with CsCl gradients [29], [30]. MCV DNA has been isolated from skin at 1.22 g/ml density using iodixanol gradients, which is in agreement with our findings [22]. The encapsidated MCV we identify in fraction 4 using uranyl acetate negative staining have the same size (∼40 nm) as similarly prepared MCV VLP but are smaller than MCV VLP (55–58 nm) visualized using phosphotungstic acid staining [7].

MCV, like other polyomaviruses, has a strictly regulated order of viral gene expression that leads to encapsidation of the viral genome. MCV LT and 57kT proteins are expressed early after MCV-HF transfection. Subsequently sT and VP1 (and presumably other virion protein components) are detectably expressed, leading to generation of self-assembling viral particles. Our study reveals an unexpected and interesting complexity for MCV gene expression. MCV LT, sT and 57kT are generated from alternatively-spliced, overlapping genes driven by the same early promoter. MCV-Rep^−^ expression for LT is initially identical to MCV-HF, indicating that the early promoter is intact, but other early proteins are diminished (57kT) or absent (sT) suggesting the possibility that viral DNA replication also regulates early TAg splicing. Late VP1 protein expression also is not detected using the MCV-Rep^−^ genome indicating that it is likely the early-to-late promoter switch also depends on viral DNA replication. This is similar to mouse polyomavirus, a close relative to MCV in the murine polyomavirus clade [5]. Well-established late lytic expression and packaging cascades for other viruses, such as herpesviruses, are also dependent on active viral DNA replication [31]. Although our results are consistent with DNA replication-dependent viral transcription, MCV-Rep^−^ is mutated with a single pentamer sequence (PS) 7/8 substitution in the MCV origin and so we cannot exclude the possibility that this point mutation also affects late promoter activity in addition to genome replication. Additional studies are needed to determine if DNA replication also regulates MCV splicing patterns.

One key factor determining MCV-HF virion production is the abundance of sT protein, which our data suggests is in turn increased in a positive feedback loop during MCV replication. When sT is coexpressed with the transfected MCV-HF genome, there is 5–6 fold increased virus production. For mouse polyomavirus, sT can signal to AP-1 and PEA3 factors to promote viral replication and late gene expression [32]. Based on SV40 studies, the most prominent role of sT involves inhibition or retargeting of substrates for the major cellular phosphatase, PP2A [33]. We previously identified MCV sT as an important accessory factor for efficient MCV DNA replication [3], but it is unknown whether this is due to PP2A or 4E-BP1 targeting, or due to targeting of our other cellular factors [15]. The Southern blot in Figure 4 reveals that MCV DNA replication is sparse and most of the replicated DNA is fragmented. This suggests that MCV genome replication, at least under conditions of plasmid transfection, is inefficient and small increases in successful MCV genome replication may have a large impact on virion replication and encapsidation. Whether the same is true during natural infection will only be determined through development of a successful MCV transmission system.

Our findings that hVam6p inhibits MCV production are unexpected and, to a degree, paradoxical. MCV LT retains a conserved hVam6p-binding domain that represses virion production. Other polyomaviruses also encode gene sequences that repress viral replication [34], which might reflect the need for these small DNA viruses to suppress virus replication to sustain a chronic, persistent infection without virion production (e.g., latency). Although the effects of hVam6p on MCV replication are clear, the mechanism by which hVam6p suppresses replication is not. Our origin replication studies in the presence and absence of hVam6p overexpression, together with LT.W209A mutations in LT coding region, show no direct effect of hVam6p on initiation of viral DNA replication. Additional studies are also needed to investigate this effect, however, since Southern blotting reveals amplified viral DNA replication for MCV-hVam6p^−^ virus compared to MCV-HF, suggesting hVam6p might be inhibitory to viral DNA replication in the context of the full viral genome.

It is noteworthy that overexpression of this vacuolar sorting protein has a profound antiviral effect on MCV replication. hVam6p possesses citron and clathrin homology domains, the latter being involved in MCV LT binding [24], that are important to its functions in the HOPS-CORVET complex as an accessory factor for endosomal fusion [35]. In yeast, the hVam6p homolog also has been reported to act as a guanine nucleotide exchange factor for Gtr1 that contributes to TORC1 activation [36]. An isoform of hVam6p, TRAP-1-like protein (TLP) regulates the balance between Smad2 and Smad3 in TGF-ß signaling [37]. Previous studies did not find either mTOR activation or TGF-ß signaling, however, to be appreciably altered by MCV LT expression [24]. In our current study, even low levels of hVam6p transfection reduce MCV-HF virus production to levels similar to the replication-deficient clone demonstrating that it is a potent factor restricting MCV replication. For the MCV-hVam6p^−^ virus, hVam6p inhibits replication in a dose-dependent manner, which may be due to either the W209A substitution incompletely disabling the hVam6p-binding site or hVam6p acting in other steps of MCV replication beyond those depending on LT. If hVam6p plays a role in inhibiting egress of MCV, this might represent a novel component of innate immunity. Tetherin, for example, is a recently-discovered innate immune component that prevents enveloped viral budding from cells [38]. Detailed analysis of hVam6p's role in antiviral responses is beyond our current study but development of a replicating MCV clone that can be genetically manipulated provides a critical reagent for use in these follow-on investigations.

Secondary MCV transmission was not detected in our study suggesting that MCV may have a tissue tropism that is not easily modeled in undifferentiated tissue culture. MCV resembles some other small DNA viruses in this way, such as JCV as well as other human tumor viruses including human papillomaviruses (HPV), hepatitis B and C viruses and KSHV. Poor MCV transmissibility could be due in part to low virus yields from our molecular clone system that might be improved with other MCV strains or cell culture settings. Cloning MCV-HF, however, provides a useful tool for these testing conditions to optimize virus yield that may ultimately allow a laboratory transmission model.

## Materials and Methods

### Cell lines and clinical samples

293 cell line (ATCC), 293FT (Invitrogen Inc.), 293TT [7], NIH3T3 (ATCC), A549 (ATCC), COS7 (ATCC) and BSC40 (ATCC) were maintained in DMEM medium supplemented with 10% FBS, penicillin and streptomycin (pen/strep). MKL-1 [13], UISO [13], BJAB (ATCC), Raji (ATCC) and peripheral blood mononuclear cell (PBMC) were cultured in RPMI 1640 medium with 10% FBS and pen/strep. MCC clinical specimens (MCC350, MCC337, MCC339, MCC344, MCC345, MCC347, MCC349 and MCC352) and PBMC sample have been described [1]. Clinical samples were collected under University of Pittsburgh Institutional Review Board (IRB) guidelines for study of human tissues.

### Characterization of MCV genomes and construct consensus MCV genomes

MCV genomes in MCC cases were directly sequenced with 13 pairs of contig primer sets as previously described [1]. Long PCR was performed to amplify the whole genome in a MCV positive PBMC sample with two primer sets (contig.1f-8r and contig.9f-1r). The consensus genome (MCV-HF) was generated from 6 tumor-type MCV genomes and 1 wild-type MCV genome using MacVector program (MacVector Inc.). The whole genome was synthesized by the DNA 2.0 Inc (Menlo Park, CA) and cloned into a Kanamycin selectable vector. The consensus MCV genome was linearized at a *BsrF*I restriction site (RCCGGY) in the VP region. A replication-defective MCV genome (MCV-Rep^−^) was mutagenesized from consensus MCV-HF with 5′-GAA AAA AAA GAG AGA GGA CTC TGA GGC TTA AGA G-3′ and 5′-CTC TTA AGC CTC AGA GTC CTC TCT CTT TTT TTT C-3′ primers, using the QuikChange Lightning Site-Directed Mutagenesis kit (Stratagene). MCV-hVam6p^−^ was generated with the primer set (W209A.S: 5′-GAA CGG ATG GCA CCG CGG AGG ATC TCT TCT GC-3′, W209A.AS: 5′- GCA GAA GAG ATC CTC CGC GGT GCC ATC CGT TC-3′) to eliminate the hVam6p binding to T antigen [24]. Plasmids containing MCV-HF, MCV-Rep^−^ or MCV-hVam6p^−^ were propagated in *E. coli* JM109 and purified using the Qiagen maxiprep kit (Valencia, CA). Linear MCV was digested out with *BsrF*I and re-ligated into circular form under low concentration of T4 ligase (1 U/µl, New England Biolabs) overnight at 16°C. Circular DNA was further digested with *Ava*I to linearize non-MCV DNA, and treated with Plasmid-Safe™ Exonuclease (Epicentre, Madison, WI) to isolate circular MCV genomes. All three genomes were sequenced confirmed.

### Nuclease-protection assay

Cells transfected with MCV-HF or MCV-Rep^−^ or MCV-hVam6p^−^ were collected and lysed with 3 freeze-thaw cycles in DPBS-Mg^2+^ buffer. After centrifugation, supernatants were treated with 250 units of benzonase (Promega) and 5 units of RNase A (Ambion) at 37°C for 4 hrs. EDTA was then added to inactivate nuclease. Proteinase K was used to digest capsid proteins at 56°C for 1 hr. The capsid-protected MCV DNA was prepared by phenol-chloroform extraction and dissolved in 50 µl TE buffer. One microliter of DNA was used for PCR quantification with VP2 primers and TaqMan probe as previously described [39].

### Immunoblotting

293 cells and stably infected 293 cells lines with LT or sT lentiviral vector (293-LT or 293-sT) were seeded in 6-well plates and transfected with 1 µg circularized MCV genomes using Lipofectamine 2000 (Invitrogen). Radio immunoprecipitation assay buffer (RIPA) was used to lyse cells. Monoclonal antibody CM2B4 [39] was used to examine LT antigen and 57kT antigen expression at 1∶2000 dilution. Monoclonal antibody CM8E6 [17] was used to detect sT antigen expression at a dilution of 1∶250. Late gene expression of VP1 was examined with monoclonal antibody CM9B2 at dilution of 1∶2000. The CM9B2 antibody (IgG2b isotype) was generated by immunizing mice with KLH-derivatized (DKGKAPLKGPQKASQKES) peptide from MCV VP1 using standard methods (Epitope Recognition Immunoreagent Core Facility, University of Alabama) and tested for reactivity to the MCV VP1 protein [7]. Tubulin (Sigma) was used to quantitate sample input at dilution of 1∶2000.

### Lentivirus production and infection

293FT cells (Invitrogen) were transfected with lentiviral construct containing MCV LT or sT antigen together with packaging plasmids, psPAX2 and pMD2.G (Addgene) in 100 mm dish by Lipofectamine 2000 (Invitrogen). 293 cells were infected with lentivirus in the presence of 1 µg/ml polybrene. At day 3 after infection, puromycin (3 µg/ml) was added, and infected cells were selected in bulk for 7 days for stably expression of MCV LT and sT antigen. Expression was confirmed by immunoblotting.

### MCV origin replication

For MCV origin replication assay, 293 cells were transfected with a plasmid containing MCV origin (Ori339(589)) [3] together with wild type (genomic T (TAg), LT) or mutant type (TAg.W209A, LT.W209A) of T antigens as well as hVam6p gene [25]. These cells were harvested at day 2 after transfection. Low molecular weight DNA was extracted with modified Hirt-extraction and Southern blot analysis was performed by method previously described [3] with a MCV origin probe (5,074–65 nt) amplified by PCR with primers: 5′-CTC GAG AGC AAT TTC ACC AAT ATT GGC C-3′ and 5′- GAT ATC TAA GCC TCT TAA GCC TCA GAG TCC-3′. To measure MCV clone replication, 2.5 µg of extracted DNA was digested overnight with 5 units of *Dpn*I and *BamH*I and subjected to Southern blotting. Same MCV origin probe was used to measure replication efficiency. The blot was analyzed by using a PhosphorImager (Typhoon 9400, GE Healthcare) and ImageJ software (National Institute of Health, USA).

### Virion purification and cell infection

At day 4 after post-transfection, cells were harvested and matured overnight with benzonase, RNase A and Plasmid-safe™ nuclease as previously described [7]. Cell lysate was separated on a 27-33-39% Optiprep (Sigma, St. Louis, MO) gradient after ultracentrifugation for 3.5 hrs at 234,000 g. Fractions were collected after puncturing the bottom of ultracentrifuge tube using a 25-G needle and stored at −80°C. Viral particles were stained with 1% uranyl acetate negative staining and observed on JEOL JEM-1011 (Tokyo, Japan) electron microscope at 80 kV and compared to self-assembling VP1-VP2 MCV virus-like particles generated as previously described [7]. In MCV infection assays, various cells (293, 293TT, UISO, A549, BJAB, Raji, BSC40 and PBMC purified from whole blood) were cultured with 4 µl ultracentrifuged fractions containing MCV virions together with or without 1 µg/ml polybrene (Sigma) treatment. For transwell experiments, 6-well transwell plates were used (Corning, New York, USA) with a 0.2 or 0.45-µm pore size polycarbonate membrane. Transfected 293 cells with MCV-HF genomes were labeled with intracellular fluorescent dye 5- (and -6)-carboxyfluorescein diacetate succinimidyl ester (CFSE) to monitor any cell contamination in transwell experiments. 293, A549 and UISO were seeded in the lower chamber to co-culture with transfected 293 cells for 6 days.
